# Sharing individual level data from observational studies and clinical trials: a perspective from NHLBI

**DOI:** 10.1186/1745-6215-14-201

**Published:** 2013-07-09

**Authors:** Sean A Coady, Elizabeth Wagner

**Affiliations:** 1 Division of Cardiovascular Sciences, National Heart, Lung, and Blood Institute, 6701 Rockledge Drive, Room 10200, Bethesda, MD, 20892, USA; 2 Division of Blood Diseases and Resources, Bethesda, USA

**Keywords:** Data sharing, Clinical trials, Cohort studies, HIPAA

## Abstract

There are numerous benefits to the research community from data sharing, and yet the open sharing of participant level data is not without potential pitfalls. In addition to the scientific community, the interests of study participants who volunteered their data must be considered, along with the interests of study investigators who expend a substantial amount of effort into the design, conduct, and analytical plans for the study. The National Heart, Lung, and Blood Institute (NHLBI) has developed a data-sharing protocol focused on balancing the interests of study participants, study investigators, and the research community with independent oversight by the NHLBI IRB. The data repository presently includes individual level data on more than 560,000 participants from 100 Institute-supported clinical trials and observational studies.

## Background

There are many potential uses of existing data: meta-analyses, applications of innovative statistical methods, replication, novel new analyses, cross-study comparisons, and sample size estimation for new studies. The benefits to the research community of data sharing are consistently heralded in the literature [1-6], and yet the open sharing of participant level data is not without potential pitfalls. First and foremost, the interests of study participants who volunteered their data must be considered along with the interests of study investigators. Participants volunteer their time and data for a variety of reasons [7,8] and maintenance of participant privacy is a primary concern [9,10]. Study investigators expend a substantial amount of effort into the design, conduct, and analytical plans for studies, and are rightfully entitled to a protected period of time with the data. Additionally, investigators that collected the data are in the best position to understand the nuances associated with the study and concerns over poorly designed secondary analyses or the motives of those requesting access to data have been expressed [11-13].

The NHLBI data repository presently includes individual level data on more than 560,000 participants from 100 Institute-supported clinical trials and observational studies. Sharing this clinical data in a manner that balances the interests of study participants, study investigators, and the research community is the subject of this commentary.

## Discussion

### A brief history of the NHLBI data repository

A formal data sharing policy, applicable to Institute-supported contract studies, was first established by the Institute in 1989 under then Director Claude L’Enfant. This first data-sharing policy timed release of data to within 3 years of ‘major publications’ and described generally the data that were to be removed from a study dataset prior to release. Although a formal data-sharing policy for contract-supported studies had been established, the availability of these datasets was not well-known, there was a lack of guidance on what process should be used to distribute datasets, and it was unclear as to what data should be made available. A revised policy, incorporating specific data release timelines, guidelines for data submission, and the data request process was developed in 1999. Since all of the data for the data repository were derived from human subjects in both ongoing and closed studies, the policy was submitted to the NHLBI Institutional Review Board (IRB) as a formal protocol and was first approved by the NHLBI IRB in 2000. The NHLBI IRB continues today in its oversight role to annually review the activities of the data repository.

### The current data-sharing policy

The data-sharing protocol has evolved over time, expanding to include grant-supported studies as well as contract-supported studies. Clarification of the common rule regarding local IRB review for secondary analysis of existing data provided additional guidance on how requests for data should be reviewed, and studies depositing data into the repository are now required to certify that the data can be shared with outside investigators.

The data repository protocol can be divided into three aspects: rules for when data will be released and what data to include, rules for submitting data to the repository, and rules governing how data will be shared.

### Data and timelines for release

The data released to the general research community through the data repository consist of all data collected during the conduct of the study including: baseline, interim visit(s), laboratory measurements, and outcome data. To minimize re-identification risks and provide reasonable protection for participant privacy, the de-identification procedures for data included in the repository generally follow the HIPAA privacy act [14], these include, but are not limited to: removal of all obvious identifiers (name, address, social security number, date of birth), removal of all dates and replacement with time intervals (that is, time since randomization or time since enrollment), and geography is generally removed. Other redactions are also encouraged such as top/bottom coding of height and weight, categorizing traits such as race/ethnicity, marital status, education, income, and so on, to reduce any re-identification risk. Sensitive items such as sexual behaviors or illicit drug use may be removed from the data when these data elements are not part of the primary focus of the study; therefore, as opposed to ‘rules’ for de-identification, the redaction process is more aptly described as following ‘guidelines’, in which considerable judgment on the part of the study is needed to maintain scientific utility and also protection of participant privacy. The goal is to minimize re-identification risk while maximizing secondary use of the redacted datasets. Over-redaction can result in a dataset with low utility.

Datasets are released following a standard timeline. Study investigators are given a protected period of time (2 years) to prepare key manuscripts from the study. In this manner the data are made available to the general research community within a reasonable time frame and provides the investigators who collected the data time to publish study results.

Data collection for clinical trials is generally predefined with specific starting and ending dates; however, observational studies may collect data over many years. Therefore, the protocol defines data release in a slightly different manner depending on the study type. Specifically, the protocol states that data from observational studies are made available: (1) 3 years after the last participant visit for a clinic exam cycle or close out date for event ascertainment; or (2) 2 years after the exam or surveillance data are made available for within study use. Ongoing observational studies will therefore tend to have periodic updates to their data. Data from clinical trials are made available 2 years after publication of the primary outcome paper or 3 years after the end of clinical activity.

It is important to note that most large clinical trials and observational studies have active publications/presentation committees that are open to collaborations with outside investigators. These studies are essentially open to immediate data-sharing using study-specific internal policies.

### Submitting data to the repository

Release of data through the data repository is required for Institute-supported contract studies and may also be required for certain large grants or cooperative agreements. The inclusion of grant-supported studies was introduced in 2005 and applies to studies reviewed and initiated after October 1, 2005. In brief, grant-supported studies with direct costs equal to or greater than $500K in any 1 year and identified as being of high programmatic interest, along with cooperative agreements with 500 or more participants are required to submit data to the data repository as part of the grant award.

All data submitted to the data repository must be consistent with the informed consent. Participants explicitly requesting that their data not be shared must be removed from all repository datasets and any other consent restrictions must be noted. For example, the informed consent for the Hemochromatosis and Iron Overload Screening Study (HEIRS) indicated that participants’ data would only be used for ‘iron-related and hereditary hemochromatosis studies’. Therefore, any requests for HEIRS study data must have an iron-related focus.

Following on the database of Genotypes and Phenotypes (dbGaP) experience, institutions submitting data to the data repository are required to certify that the data can be shared. In brief, the certification affirms to the NHLBI that the informed consent explicitly permits sharing data with other investigators, or the depositing Institution’s IRB has approved the sharing of the participants’ data.

### Accessing data in the repository

Applications for study datasets in the Data Repository are done online at http://www.biolincc.nhlbi.nih.gov. The website is maintained by the NHLBI Biologic Specimen and Data Repository Information Coordinating Center (BioLINCC) program. The online application for study datasets include: (1) a description of the research project; (2) local IRB review or institutional certification of exemption from IRB review; and (3) agreeing to the terms and conditions of a data use agreement (termed a Research Materials Distribution Agreement (RMDA) since both biospecimens and data can be obtained from the NHLBI repositories). Terms of the RMDA include provisions limiting use of the data to 3 years, prohibits transfer of the data to another party, requires appropriate computer security measures, and specifies the acknowledgment to be used in manuscripts. Both the investigator and the Institution are required to sign and agree to the terms of the RMDA. Applicants are not subject to a scientific review, but instead an administrative review is conducted to ensure the project is within the spirit and intent of the data agreement. Likewise, there is no requirement for screening manuscripts prior to submission to a journal. The absence of a scientific review or pre-submission review of manuscripts avoids any perception of blocking access or controlling research; however, this does allow for the possibility of misleading or incorrect analyses to be published. The possibility of intentionally misleading analyses is mitigated to some degree through the open nature of the repository in that replication is possible with the identical dataset. Avoiding unintentionally incorrect analyses is far more difficult and is largely only correctable post publication.

A summary of the clinical data and biospecimen resources available are provided in Additional file 1: Table S1.

## Conclusion

A collaborative approach, utilizing the experience and expertise of study investigators, is the most productive method of data-sharing for ensuring high quality manuscripts. The NHLBI data repository fulfills the niche of providing opportunities for secondary analysis of existing data when collaborations are either unfeasible, or not desired. Participant data are released, after study investigators have had a 2-year protected period of time, in a manner consistent with the informed consent, and is generally redacted to meet or exceed HIPAA requirements to maintain participant privacy.

The NHLBI has made a commitment to the sharing of research resources to the widest possible audience to maximize the value of Institute-supported studies. Through a website portal, the Institute financially supports the infrastructure to efficiently process, communicate information, and distribute to the research community data from more 560,000 study participants and 4.6 million biological samples.

## Supplementary Material

Additional file 1: Table S1 Observational studies, clinical trials, and transfusion medicine studies with data and/or samples in the NHLBI data and biospecimen repositories. Study acronym and number of participants are in parentheses. **Bolded** studies have samples in the biorepository and the footnotes indicate stored biospecimen types. Click here for file
