# Supplementary material for: Sharing individual level data from observational studies and clinical trials: a perspective from NHLBI
Source: Trials. 2013 Jul 9;14:201. doi: 10.1186/1745-6215-14-201 (PMC3750470; doi:10.1186/1745-6215-14-201)
Supplement: Additional file 1: Table S1 — Observational studies, clinical trials, and transfusion medicine studies with data and/or samples in the NHLBI data and biospecimen repositories. Study acronym and number of participants are in parentheses. Bolded studies have samples in the biorepository and the footnotes indicate stored biospecimen types. [file 1745-6215-14-201-S1.docx]

Table 1. Observational studies, clinical trials, and transfusion medicine studies with data and/or samples in the NHLBI data and biospecimen repositories. Study acronym and number of participants are in parentheses. **Bolded** studies have samples in the biorepository and the footnotes indicate stored biospecimen types.

|  |  |  |
| --- | --- | --- |
| Observational Studies | | |
|  | Population Studies | |
|  |  | Atherosclerosis Risk in Communities (ARIC, 15732); Cardiovascular Health Study (CHS, 5795); Coronary Artery Risk Development in Young Adults (CARDIA, 5113); Framingham Heart Study-Original Cohort (5079), Framingham Heart Study-Offspring (5013); Framingham Generation 3 cohort (4078), **Honolulu Heart Program (HHP, 8006)^abd^**; Lipid Research Clinics Prevalence Study (LRC-PS, 60495); Multi-Ethnic Study of Atherosclerosis (MESA, 6814); Puerto Rico Heart Health Program (PRHHP, 9824); Women’s Health Initiative Observational Study (WHI-OS, 93676); Jackson Heart Study (JHS, 2063) |
|  | Selected Patient Populations | |
|  |  | **A Case-Control Etiologic Study of Sarcoidosis (ACCESS, 1404)^bde^**; Alpha_1_ Antitrypsin Deficiency Registry (1129); **Cooperative Study of Sickle Cell Disease (CSSCD, 4085)^a^**; Cord Blood Transplant Study (COBLT, 364); **Hemochromatosis and Iron Overload Study (HEIRS, 2319)^abdgi^**; Primary Pulmonary Hypertension Registry (PPH, 317); Retrovirus in Infected Donors (REDS-HTLV, 1474); Women’s Ischemia Syndrome Evaluation (WISE, 1024); **Non-A, Non-B Hepatitis long term followup (NANB, 1552)^a^**; **Pediatric Pulmonary and Cardiovascular Complications of Vertically Transmitted HIV Infection (P2C2, 816)^a^**; Retrovirus Epidemiology Donor Study II-Donation and Deferral Database (CORE, 700K); **Retrovirus Epidemiology Donor Study II-Leukocyte Antibodies Prevalence Study (LAPS, 7900)^ab^; Retrovirus Epidemiology Donor Study II-Donor Iron Status Evaluation Study (RISE, 1537)^bd^** |
|  | Adolescents | |
|  |  | Bogalusa Heart Study (BHS, 11796); **NHLBI Growth and Health Study (NGHS, 2379)^a^** |
|  | | |
| Clinical Trials | | |
|  | Asthma Clinical Research Network Trials | |
|  |  | Beta Agonist in Mild Asthma (BAGS, 255); Colchicine in Moderate Asthma (CIMA, 71); Salmeterol or Corticosteroids and Salmeterol with and without Inhaled Corticosteroids (SOCS/SLIC, 339); Measuring Inhaled Corticosteroids Efficacy (MICE, 30); Dose of Inhaled Corticosteroids with Equisystemic Effects (DICE, 156); Beta Adrenergic Response by Genotype (BARGE, 94); Improving Asthma Control (IMPACT, 225); Predicting Response to Inhaled Corticosteroid Efficacy (PRICE, 72); Smoking Modulates Outcomes of Glucocorticoid Therapy (SMOG, 83); Asthma Clinical Research Network Salmeterol and Leukotriene Modifiers vs. Salmeterol and ICS Treatment (SLiMSIT, 192) |
|  | Acute Respiratory Distress Network Trials | |
|  |  | **Lower versus higher tidal volume, lisofylline treatment and ketoconazole treatment (ARMA/KARMA/LARMA, 902)^abh^; Efficacy of Corticosteroids as Resuce Therapy for the Late Phase of Acute Respiratory Distress Syndrome (LaSRS, 180)^bef^; Assessment of low tidal volume and elevated end-expiratory pressure to obviate lung injury (ALVEOLI, 550)^b^; Fluid and Catheter Treatment Trial (FACTT, 1000)^ab^** |
|  | Community Interventions | |
|  |  | Rapid Early Action for Coronary Treatment Study (REACT, 4389); Public Access Defibrillation (PAD) |
|  | Hypertension Treatment/Blood Pressure control | |
|  |  | **Dietary Approaches to Stop Hypertension Study (DASH, 412) ^abh^; Dietary Approaches to Stop Hypertension - Sodium Study (DASH-Sodium, 459)^abh^**; **Trial of lifestyle Interventions for Blood Pressure Control (PREMIER, 810)^abhi^**; Trials of Hypertension Prevention, Phases I and II (TOHP, 4564); Prevention and Treatment of Hypertension Study (PATHS, 641) |
|  | Primary Prevention | |
|  |  | Multiple Risk Factor Intervention Trial (MRFIT, 12866); Activity Counseling Trial (ACT, 3915); Lipids Research Clinics Coronary Primary Prevention Trial (LRC-CPPT, 3806) |
|  | Secondary Prevention | |
|  |  | Aspirin Myocardial Infarction Study (AMIS, 4524) ; Asymptomatic Cardiac Ischemia Pilot (ACIP, 1820); Beta-Blocker Evaluation of Survival Trial (BEST, 2708); Beta-Blocker Heart Attack Trial (BHAT, 3837); Cardiac Arrhythmia Suppression Trial (CAST, 3549); Coronary Artery Surgery Study (CASS, 27279); Digitalis Investigation Group (DIG, 7788) ; Magnesium in Coronaries Study (MAGIC, 6213); Studies of Left Ventricular Dysfunction (SOLVD, 6795); Thrombolysis in Myocardial Infarction Study (TIMI II, 3339 and TIMI III, 1473); **Prevention of Events with Angiotensin Converting Enzyme Inhibition (PEACE, 8290)^abh^**; Psychophysiological Investigation of Myocardial Ischemia (PIMI, 196); Post Coronary Artery Bypass Graft Study (PostCABG, 1351); Evaluation Study of Congestive Heart Failure and Pulmonary Artery Catheterization Effectiveness (ESCAPE, 433); Atrial Fibrillation Follow-up Investigation of Rhythm Management (AFFIRM, 4061); Women's Angiographic Vitamin and Estrogen Trial (WAVE, 423); Enhancing Recovery in Coronary Heart Disease Patients (ENRICHD, 2481); **Optimal Macronutrient Intake Trial to Prevent Heart Disease (OMNIHeart, 164)^abh^**; Antiarrhythmics Versus Implantable Defibrillators (AVID) |
|  | Primary/Secondary Prevention | |
|  |  | Anti-hypertensive and Lipid Lowering Trial (ALLHAT, 42418); Systolic Hypertension in the Elderly Program (SHEP, 4736); Women's Health Initiative: Clinical Trials (WHI-CT, 68132); Hypertension Detection and Follow-up Program (HDFP, 10940) |
|  | Diagnostic | |
|  |  | Prospective Investigation of Pulmonary Embolism Diagnosis (PIOPED, 1487) |
|  | Lung Function/Respiratory | |
|  |  | Intermittent Positive Pressure Breathing (IPPB, 3218); Lung Health Study (LHS, 5887); National Emphysema Treatment Trial (NETT, 1218); |
|  | Other Treatment trials | |
|  |  | **Multicenter Study of Hydroxyurea (MSH, 299)^adg^**; Raynaud's Treatment Study (RTS, 313); **Weight Loss Management (WLM, 1443)^ab^**; Blood and Marrow Transplant Clinical Trials Network (BMT CTN) Comparison of Fluconazole Versus Voriconazole to Treat Fungal Infections in Individuals Receiving Blood and Marrow Transplants (BMTCTN0101, 600) |
|  | Blood transfusion/blood products | |
|  |  | **Trial to Reduce Alloimmunization to Platelets (TRAP, 603)^a^**; T-Cell Depletion in Unrelated Donor Marrow Study (TCD, 410); Viral Activation Transfusion Study (VATS, 814) |
|  | Trials in adolescents | |
|  |  | Childhood Asthma Management Program (CAMP and CAMPCS, 1041); Dietary Intervention Study in Children (DISC, 663); High Frequency Ventilation in Premature Infants Study (HIFI, 673); Trial of Activity in Adolescent Girls (TAAG, 2640) |
|  | | |
| Transfusion Medicine (no clinical data) | | |
|  |  | **Retrovirus Epidemiology Donor Study (REDS) Special Repository (SR) Collections^abcjk^; Transfusion-Transmitted Viruses Study (TTVS)^a^; Human Herpes Virus 8 (HHV-8) Special Collection from the General Leukocyte/Plasma Repository^bc^; REDS Allogeneic Donor and Recipient Repository (RADAR)^abc^; NHLBI Umbilical Cord Blood Unit Collection^l^; General Serum Repository (GSR) and General Leukocyte/Plasma Repository (GLPR)^abc^** |
|  |  |  |

^a^ serum ^e^ bronchoaveolar lavage ^i^ buffy coat

^b^ plasma ^f^ leukocytes ^j^ peripheral BL mono cells

^c^ whole blood ^g^ lymphocytes ^k^ platelets

^d^ DNA ^h^ urine ^l^ cord blood
